# Effects of an Early Exercise Program with Cryotherapy on Range of Motion, Pain, Swelling, and Gait in Patients with Total Knee Arthroplasty: A Randomized Controlled Trial

**DOI:** 10.3390/jcm13051420

**Published:** 2024-02-29

**Authors:** Bomi Lee, Doyoo Yoon, Jongeun Yim

**Affiliations:** Department of Physical Therapy, The Graduate School of Sahmyook University, Seoul 01795, Republic of Korea; bomilee9104@gmail.com (B.L.); zamgo2020@gmail.com (D.Y.)

**Keywords:** cryotherapy, gait, pain, range of motion, total knee arthroplasty

## Abstract

**Background**: This study aimed to investigate the effects of cryotherapy on range of motion, pain, swelling, and gait in patients who underwent total knee arthroplasty. **Methods**: Forty-three patients who underwent TKA (total knee arthroplasty) and met the inclusion criteria were randomly divided into two groups. The experimental (n = 21) and control (n = 22) groups underwent cryotherapy and non-cryotherapy treatments, respectively, six times a week for two weeks, and once each before and after exercise for 3 min. Both groups followed a similar initial rehabilitation exercise program using a continuous passive motion device. **Results**: The results showed a significant difference in knee flexion range of motion, pain, edema, and 10 MWT comparisons from pre- to post-test (*p* < 0.001). The above values were also significantly different in the comparison between the two groups (*p* < 0.05). **Conclusions**: Therefore, this study confirmed that an initial rehabilitation exercise program accompanied by cryotherapy could be an effective intervention method for range of motion, pain, edema, and walking in patients undergoing total knee arthroplasty.

## 1. Introduction

As individuals age, they are reported to be more affected by osteoarthritis, which is the leading cause of joint degeneration [1]. Osteoarthritis is becoming the most common joint disease worldwide, affecting approximately 10% of men and 18% of women >60 years of age [2]. It can be a major cause of pain, joint deformity, decreased quality of life, and physical dysfunction. Among the different joints, knee osteoarthritis is the most common degenerative arthritis because it is a weight-bearing joint [3,4].

Total knee arthroplasty (TKA) is an effective treatment to relieve pain, correct joint deformity, and normalize the range of motion in patients with degenerative knee osteoarthritis who have failed conservative treatment with medication or physical therapy [5,6]. Total knee arthroplasty has been reported to be a superior surgical procedure that improves patients’ quality of life, and the global demand for TKA has increased dramatically over the past 50 years since the 1970s, with an expected 85% increase in TKA procedures (1.26 million) by 2030 [7,8]. However, in many cases, if rehabilitation after TKA is not performed properly, the patient may not be able to exercise independently due to daily discomfort, severe pain, and swelling, and the joint may become increasingly stiff and rigid, causing various complications, such as leg muscle loss, capsular contracture, joint soft tissue hardening, and delayed rehabilitation [9,10].

After TKA, patients with these problems have lower levels of function over the course of a year compared to non-operated older adults, and they have greater difficulty performing functional movements, including a 40% decrease in quadriceps strength, an 18% decrease in walking speed, and a 51% decrease in stair climbing speed [9]. Furthermore, postoperative knee flexion contractures alter balance in the standing posture due to postural sway and changes in the mean position of the foot pressure center [11]. Restricted knee joint movement can cause issues with other body parts and weight bearing. A limitation in knee flexion increases posterior pelvic tilt in standing and overall pelvic tilt during walking, directing the trunk to the side with joint restriction. Additionally, limited knee extension on one side increases weight bearing on the opposite leg, leading to asymmetrical weight distribution on both legs [6]. Consequently, rehabilitation is recommended as early as possible after TKA to achieve optimal surgical outcomes and improve the functional performance of the knee joint [12]. Initial postoperative rehabilitation exercises include strength training, continuous passive motion (CPM), and passive range of motion exercises, while other interventions include balance, proprioception, and water resistance exercise [13].

However, it is well known that the pain experienced by patients after TKA can be so severe that they are unable to concentrate during exercise, and muscle weakness secondary to pain is a barrier to early rehabilitation [14]. Physical therapists should implement interventions to help patients with early post-arthroplasty pain and improve their physical function, such as strength and range of motion, which can lead to long-term benefits for future patients, such as improved gait speed [15]. Current interventions include rest, ice, compression, and elevation to reduce edema, bleeding, and pain [16]. Among these interventions, cryotherapy has been widely used in postoperative care because it is cost-effective and relatively safe compared to other interventions in clinical practice [17].

The goal of ice packs, which are usually applied in physical therapy offices, is to reduce the temperature in the joint cavity, reduce blood circulation in soft and bone tissues, and reduce metabolism in the knee joint. After 25 min of application, which is when the treatment is most effective, ice packs have been shown to produce strong anti-inflammatory effects in similar inflammatory conditions, such as back pain, acute bursitis, and tendonitis. This is achieved through changes and reductions in pain transmission, resulting in the soothing of acute pain [18,19]. The mechanism and effects of cold treatment are as follows. Firstly, it has a hemodynamic effect which is effective for acute inflammation due to the reflex mechanism. This is achieved through immediate cutaneous vasoconstriction followed by reflex vasodilation due to direct vascular smooth muscle contraction. The substance has two effects. Secondly, it has a neuromuscular effect due to the reduction in excitatory firing frequency of the musculocutaneous and Golgi tendon organs, nerve conduction velocity, muscle fatigue, and muscle stiffness. Thirdly, it has effects on joint and connective tissue by reducing collagenase activity and elongating tendons through decreasing intra-articular temperature [20].

In recent years, cryotherapy, which involves cooling air nitrogen to −30 °C and applying it to an affected area for a short period of time, has gained in popularity [21]. The air cooling method of cryotherapy absorbs heat through a liquid refrigerant in the evaporator of the chiller, and refrigeration occurs when the refrigerant changes from liquid to gas. At this time, the refrigerant is compressed, and due to high pressure and temperature, heat is released to the outside; simultaneously, it is a treatment device that emits cold air [22].

However, cold treatment can have side effects due to long treatment time, inconsistent cold temperatures, and direct contact. Nevertheless, cryotherapy has the advantage of achieving similar effects within a short treatment time of a few minutes, and it is relatively safe, owing to its non-contact method; therefore, it is easy to apply and highly efficient without any special side effects. Therefore, this study aimed to investigate the effects of cryotherapy on range of motion, pain, edema, and gait in patients undergoing TKA and to suggest effective intervention methods.

## 2. Materials and Methods

### 2.1. Participants

This study included hospitalized patients who underwent total knee replacement at the Bucheon City Orthopedic Hospital in Gyeonggi-do. The selection of participants was conducted using the G-power program (IBM Inc., Armonk, NY, USA); when the POWER value was set to 0.80 and the effect size (Cohen’s) was selected as 0.90, the calculated number of participants was 42, and, considering a dropout rate, the total number of participants was 46.

All the participants were pretested before starting the study, and the 46 patients were randomly divided into 23 experimental patients in a cryotherapy treatment group and 23 control patients in a sham treatment group. Three patients (two in the experimental group and one in the control group) dropped out because of physical problems, early discharge, and personal reasons. Finally, 21 and 22 patients in the experimental and control groups participated in the study, respectively. Inclusion criteria for the study were patients who underwent total knee replacement surgery due to degenerative arthritis, patients with no neurological impairment that would affect their ability to perform the exercise program, patients with no postoperative inflammation or other adverse events, and patients aged ≥60 years who could adequately understand the study methods and objectives. Exclusion criteria for the study included patients with back or lower extremity damage or surgery in the past two years, patients with a history of surgery on the knee joint prior to total knee replacement, and patients with circulatory disturbances or allergies to cold. All consenting participants eligible for TKR were considered for inclusion with the exception of those experiencing or suspected of hematologic and coagulation disorders. If the patient was suspected of having a blood clot, aspirin or persantin was prescribed. Patients took narcotic pain medication for three days immediately following surgery, followed by acetaminophen and anti-inflammatory medication.

The purpose, procedures, and expected effects of the experiment were explained in detail to the participants, and only those who signed the consent form were selected for the study. The study was approved by the Institutional Review Board of Sahmyook University (approval number: 2-1040781-A-N-012021079HR). This study was registered with the Clinical Research Information Service (CRIS) (KCT0009200).

### 2.2. Procedure

**Figure 1:**
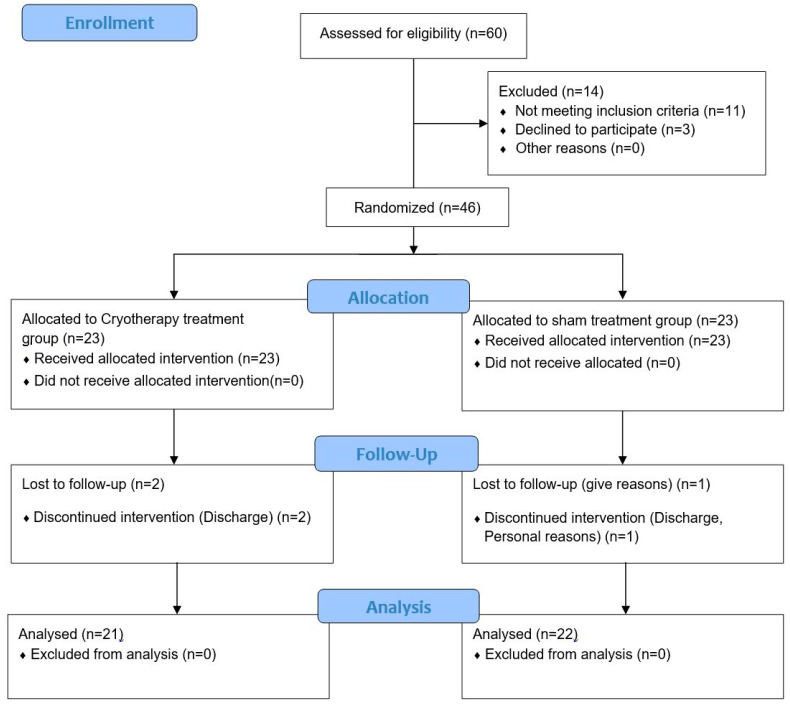
Study flow diagram.

All patients in both groups underwent medial parapatellar arthrotomy with tourniquet use for the TKA. A pretest was performed by physical therapists on all the study participants before starting the experiment. The 46 patients were randomly divided into 23 experimental patients in the cryotherapy treatment group and 23 control patients in the sham treatment group. Three patients (two in the experimental group and one in the control group) dropped out, owing to physical problems, early discharge, and personal reasons. Finally, 21 and 22 patients in the experimental and control groups, respectively, participated in the study. The pre- and post-test assessments included range of motion, pain, edema, and gait. Goniometers (Goniometers, Fabrication Enterprises Inc., White Plains, NY, USA) were used to measure the participants’ range of motion, the visual analog scale (VAS) was used to measure pain levels, a tape measure (Rollfix, Hoechst Mass, Frankfurt, Germany) was used to measure edema, and the 10 m walking test (10 MWT) was used to measure gait. The study was initiated on postoperative day three of the total knee replacement. The experimental group underwent cryotherapy treatment twice for 3 min, once before and after the exercise, and the initial rehabilitation exercise lasted for 30 min, six times a week for two weeks, for a total of 12 sessions by physical therapists. The control group received the sham treatment for 3 min twice, once before and after the exercise. Additionally, the experimental and control groups performed CPM (continuous passive movement) once daily for 30 min, six times a week for two weeks, for a total of 12 sessions (Figure 1).

### 2.3. Intervention

#### 2.3.1. Cryotherapy Treatment

Cryotherapy was performed by applying cryogenic liquid carbon dioxide gas at −78 °C, approximately 10 cm perpendicular to the joint line of the knee joint, with care to avoid concentrated spraying to prevent skin frostbite. The cryotherapy cycle was applied twice for 3 min, once before and after the initial rehabilitation exercise, for 12 days. The treatment duration was 3 min, based on previous studies that reported that treatment can be applied once or twice a day, and, depending on the treatment area, it can be 1–5 min [23,24]. The sham treatment was applied in the same way as the cryotherapy treatment, but without producing cryogenic liquid carbon dioxide gas.

#### 2.3.2. Initial Rehabilitation Program

The experimental and control groups completed daily 30 min exercise sessions, once a day, for a total of 12 sessions over a two-week period. The initial rehabilitation program was based on previous studies and began three days after the drainage tube was removed following surgery. The level of difficulty was adjusted to suit the early postoperative period. Tailored to the early postoperative period, the exercise program was carefully calibrated to align with each patient’s recovery progress. The program included a variety of exercises, starting with basic movements and gradually increasing in difficulty: knee joint active-assistive range of motion, straight leg raise exercise, quadriceps setting exercise, ankle pumping, and leg extension. The postoperative rehabilitation program included a knee-pressing exercise comprising 5 sets of 12 repetitions, each lasting 5 s, with a 30 s rest between sets. This approach prioritizes patient safety and effective progression. It also accommodates and supports individual recovery pathways [25].

#### 2.3.3. Continuous Passive Movement

The experimental and control groups received CPM for 30 min daily over a period of two weeks, comprising 12 sessions in total, beginning on the third day following total knee arthroplasty. The CPM device was installed by a physical therapist, and the procedure was standardized. Identical installations were performed for both groups: subjects lay supine in their bed, and the CPM device was placed under the affected leg with the knee extended. For stability, one strap surrounded the subject’s thigh, another strap surrounded the subject’s lower leg, and the apparatus was prevented from sliding down by the edge of the bed. The range of knee flexion was recorded by setting the range of flexion joint motion according to the patient’s tolerance, starting at 0° and gradually increasing the range of flexion according to the patient’s condition.

### 2.4. Outcome Measures

#### 2.4.1. Joint Range of Motion Measurement

A goniometer (Goniometer; Baseline, Little Rock, AR, USA) was used to measure the range of flexion and extension of the knee joint. Flexion was measured with the patient in the supine position, with both legs fully extended and attached to the floor. The axis was positioned at the lateral femoral condyle of the knee joint, the stabilizer at the greater trochanter, and the movers at the line connecting the fibula’s lateral malleolus. The flexion angle was measured at the point of pain and at the maximum angle of the joint, and the average value was obtained from three measurements [26]. The knee joint extension was measured by placing a towel under the femur to measure the maximum extension without any force from the experimenter in the prone position. The goniometer was placed at the same reference point as the flexion, the passive extension was measured three times in total, and the average value was used [27].

#### 2.4.2. Pain Measurement

The patient’s pain was measured using VAS in the form of a subjective report, with the left end of a 10 cm horizontal line at 0 cm (no pain) and the right end at 10 cm (very severe pain), with the question “How much pain do you feel right now?” Pain was scored on a scale of 0–10, with 0 cm as 0, and 10 cm as 10. In this case, the pain assessment described the pain felt by the participant at rest in daily life; the reliability of the VAS is 0.62, and the validity was 0.75 [28].

#### 2.4.3. Edema Measurement

In this study, edema was defined as a change in lower extremity circumference. Previous studies have shown that edema measurement methods that measure lower extremity circumference are highly correlated with ultrasound and bioimpedance measurements. A tape measure (Rollfix, Hoechst Mass, Sulzbach, Germany) was used to measure the circumference, and after removing the postoperative bandage, an area corresponding to 5 cm and 10 cm above the knee joint line was marked in the supine position, and the average value of three repeated measurements of the diameter of the area was used [29,30].

#### 2.4.4. The 10 MWT

Considering that the participants were early TKA patients and that the study was performed in a clinical environment, the 10 MWT was used as an assessment tool to evaluate walking speed among walking abilities and was conducted by referring to previous studies. In the 10 MWT, the participant was instructed to walk at a comfortable speed along a straight line, extending the distance 2 m before and after the 10 m distance to avoid the evaluation of deceleration and acceleration of walking speed. The therapist began measuring with a second watch when the initial 2 m point was passed and ended the measurement at the last 2 m point. A brace could be used, depending on the participant’s condition, and a post-assessment was conducted using the same brace [31].

### 2.5. Statistical Analysis

Statistical analyses were performed using SPSS version 21.0. The normality of the variables was checked using the Shapiro–Wilk test to confirm that all variables were normally distributed. To examine sex differences in the general characteristics of the study participants, a chi-square test was used. Age, height, weight, and body mass index (BMI) were presented as means and standard deviations using an independent *t*-test.

An independent *t*-test was used to determine differences between the groups, and a paired *t*-test was used to compare pre- and post-intervention differences within each group. All statistical significance levels were set at 0.05.

## 3. Results

The study included 21 and 22 patients in the cryotherapy and control groups, respectively; the general characteristics of both groups were homogeneous. Age, height, weight, and BMI were compared between the two groups, but there were no significant differences (Table 1).

### 3.1. Knee Joint Rage of Motion

The knee joint flexion angle of the experimental group significantly increased from 52.24° to 115.69° before and after the intervention, respectively (*p* < 0.05), whereas that of the control group significantly increased from 62.73° to 100.75° before and after the intervention, respectively (*p* < 0.05). In the comparison of the differences between the two groups, according to the intervention method, a significant difference was observed in the experimental group (*p* < 0.05). The experimental group showed a significant decrease in the knee joint extension angle from 11.76° to 5.24° before and after the intervention, respectively (*p* < 0.05) (Table 2). The control group showed a significant decrease in the knee glenoid angle from 12.80° to 6.23° before and after the intervention, respectively (*p* < 0.05). A comparison of the differences between the two groups, according to the intervention method, revealed no significant differences in the experimental group (Table 2).

### 3.2. Pain

The experimental group showed a significant decrease in VAS scores from 5.05 to 2.00 points before and after the intervention, respectively (*p* < 0.05), while the control group showed a significant decrease from 4.90 to 3.27 points before and after the intervention, respectively (*p* < 0.05). When comparing the differences between the two groups, according to the intervention method, a significant difference was found in the experimental group (*p* < 0.05) (Table 3).

### 3.3. Edema

The 5 cm circumference above the knee in the experimental group was 42.09 cm and 38.40 cm before and after the intervention, respectively (*p* < 0.05), and in the control group, it was 41.81 cm and 40.14 cm before and after the intervention, respectively (*p* < 0.05). When comparing the differences between the two groups, according to the intervention method, a significant difference was found in the experimental group (*p* < 0.05) (Table 3). The 10 cm circumference above the knee in the experimental group was 44.36 cm and 40.68 cm before and after the intervention, respectively (*p* < 0.05), and in the control group, it was 43.54 cm and 41.87 cm before and after the intervention, respectively (*p* < 0.05). When comparing the differences between the two groups, according to the intervention method, a significant difference was found in the experimental group (*p* < 0.05) (Table 4).

### 3.4. Gait

The walking speed of the experimental group was 20.85 m/s and 11.79 m/s before and after the intervention, respectively (*p* < 0.05), and in the control group, it was 19.52 m/s and 14.8 m/s before and after the intervention, respectively (*p* < 0.05). When comparing the differences between the two groups, according to the intervention method, a significant difference was found in the experimental group (*p* < 0.05) (Table 5).

## 4. Discussion

This study investigated the effects of a two-week early rehabilitation program with cryotherapy on range of motion, pain, edema, and gait in 43 patients who underwent TKA. The results of the two-week study showed that the initial rehabilitation program with cryotherapy significantly improved knee flexion range of motion, pain, edema, and gait when comparing the groups. This suggests that cooling may have a positive effect on patients who undergo early postoperative knee replacement. In the early postoperative rehabilitation phase, range of motion exercises can effectively reduce pain, joint effusion, and movement-limiting scar tissues. Among interventions to increase range of motion, cryotherapy has been reported to improve range of motion by reducing muscle spasms, which increases stretching and decreases muscle contraction, along with improving pain relief [32,33]. Kullenberg et al. (2006) found a significant difference in knee flexion angle from 50.4° to 98.9° after three weeks in an experimental group that received cold therapy after total knee replacement [34]. In this study, we assessed the effect of cryotherapy on the range of joint motion of the experimental group, and a significant increase was found in both the experimental and control groups (*p* < 0.05). In the comparison of differences between the groups, the experimental group with cryotherapy was significantly different from the control group with the sham treatment (*p* < 0.05). The flexion angle of the experimental group was consistent with Rowe et al.’s (2000) recommendation of a minimum of 110° of knee joint flexion [35]. The results of this study suggest that cold therapy is an effective intervention that reduces postoperative pain, restores adequate range of motion, and maximizes flexion, which are the primary goals of TKA [36]. Kullenberg et al. (2006) reported that early range of motion gains through cooling can lead to better coordination of the lower extremity muscles and improved knee joint movement, which can lead to faster rehabilitation. The results of this study suggest that cold treatment is an effective intervention for improving knee range of motion in TKA patients [34].

In our study, the flexion angle increased more than the extension angle, which may be due to the fact that Korean patients require a larger flexion angle as an inconvenience of flexion restriction, so we focused more on flexion motion rehabilitation to meet the needs of patients [14], and, based on previous studies, cryotherapy was performed in a flexion position, which did not have a significant effect on the extension angle between groups. And the joint capsule pattern of the knee is more restrictive of flexion than of extension, which may explain why there was no significant difference in the angle of extension with cold therapy.

During total knee arthroplasty, patients may experience moderate to severe pain, which can result in high analgesic requirements. Additionally, patients often report severe postoperative pain that can hinder their ability to perform CPM, which is crucial for rehabilitation [37,38]. This study used cryotherapy, which is one of the methods of Korean cold treatment; this intervention is performed by spraying carbon dioxide gas at high pressure, and it is said to reduce the painful area by applying a very low temperature and a strong pressure [39]. The results of the present study showed a significant reduction in both the experimental group, which received cryotherapy treatment, and the control group, which received the sham treatment (*p* < 0.05). In the between-group comparison, the cryotherapy treatment group was significantly different from the sham control group (*p* < 0.05). This is consistent with a previous study by Karaduman et al. (2019), who found that cooling reduced pain at 6 h, 24 h, and 48 h postoperatively in patients who underwent knee arthroscopic surgery, suggesting that postoperative cooling is effective in reducing pain, length of hospital stay, and analgesic use [40]. The results indicate that cooling, which stimulates subcutaneous cold receptors, blocks the transmission of pain impulses along the spinal cord and cerebral cortex. Pain reduction is achieved by increasing pain thresholds and blunting pain sensation. Currently, cryotherapy is used more effectively than conventional cold therapy [39,41], and Daniel et al. (1994) reported that pain relief through cold treatment can manifest as a decrease in soft tissue swelling, decrease in muscle spasm, and increase in pain threshold [42]. Additionally, even after a long time has passed since TKA, severe pain negatively affects daily life recovery; therefore, it is necessary to strive for rapid recovery and pain reduction in the early postoperative period; cold treatment, which is an effective intervention method, will help.

Persistent edema following surgery or trauma interferes with tissue growth factors, delays tissue regeneration, and stimulates pain receptors to exert pressure [43]. Mora et al. (2002) used a pulsatile cold compression cuff in patients after ankle fracture surgery. They observed a significant reduction in ankle circumference in an experimental group compared to a control group without cryotherapy at 24 h, 48 h, and 72 h after surgery [44]. Williamson et al. (1998) found that cold treatment significantly reduced edema and bleeding after TKA [45]. In this study, the circumference of the lower extremity edema after TKA was measured at 5 cm and 10 cm above the knee joint line, referring to previous studies on cold therapy. The results of the study showed a significant decrease in both the experimental and control groups at 5 cm and 10 cm above the knee joint line (*p* < 0.05). In the intergroup comparison, the experimental group with cryotherapy was significantly different from the control group with the sham treatment (*p* < 0.05). The significant difference between the experimental and control groups in the intergroup comparison was due to the decrease in blood flow caused by the cold treatment. This decrease was attributed to vasoconstriction and decreased capillary permeability. The cold treatment was applied as an appropriate intervention to control edema by inhibiting the movement of water from capillaries to interstitial tissues and controlling bleeding and fluid loss after acute trauma [46].

Many problems are observed in the early postoperative period after TKA, including pain and abnormal gait due to functional limitations caused by a weakened lower extremity joint [47]. To solve this problem, Okoro et al. (2019) reported that cryotherapy could positively affect early gait rehabilitation in early total hip arthroplasty patients [48]. Therefore, this study aimed to investigate the effect of cryotherapy on walking speed based on previous studies and measured walking time using a 10 MWT. A significant reduction was observed in both the experimental and control groups (*p* < 0.05). In the intergroup comparison, the experimental group with cryotherapy was significantly different from the control group with the sham treatment (*p* < 0.05). Additionally, a 6 min walking test using cooling after TKA in a previous study showed that the experimental group walked a longer distance than the control group [49]. The study measured the change in walking speed over a distance of 10 m. The results indicate a significant difference between the experimental group, which received cooling treatment, and the control group, which received the sham treatment. This difference is primarily attributed to the pain reduction achieved through cooling treatment. It is known that the time required to return to daily life and independent walking after TKA is 2–3 months, but the speed of recovery varies greatly, depending on how the rehabilitation is performed; it is thought that initial cooling treatment can improve walking speed in patients who undergo TKA and have a positive effect on maintaining walking ability and independent walking [50].

This study aimed to investigate the effects of an early rehabilitation program with cooling on joint range of motion, pain, edema, and gait in patients who underwent TKA. We found that cooling is an effective intervention for patients with TKA and can be used efficiently without specific side effects.

This study has some limitations. First, the study had a small number of participants, which makes it difficult to generalize the results. Second, the study determined the short-term effect of the intervention, which was two weeks; however, the long-term effect could not be determined. Third, the study could not control all daily activities outside of the intervention time during the hospitalization period; therefore, each variable could be affected by the study. In future studies, it will be necessary to increase the number of participants and the duration of intervention, with appropriate control of participants.

## 5. Conclusions

In conclusion, this study examined the effects of an early rehabilitation program with cryotherapy on patients who underwent TKA. The results showed that the initial rehabilitation program with cryotherapy significantly improved knee flexion range of motion, pain reduction, edema control, and gait compared to a control group. Cryotherapy may have a positive impact on early postoperative knee replacement patients by enhancing range of motion, reducing pain, and aiding in the restoration of functional gait. Furthermore, cryotherapy has been found to be effective in alleviating postoperative pain, possibly by increasing pain thresholds and blunting pain sensation. It also helps to reduce edema by inducing vasoconstriction and decreasing capillary permeability. Furthermore, cryotherapy had a positive impact on walking speed, which can enhance rehabilitation outcomes and maintain walking ability in patients who have undergone TKA.

## Figures and Tables

**Table 1:** General characteristics of the subjects (n = 43).

ROM	CRYO (n = 21)	Non-CRYO (n = 22)	t (*p*)
Gender (male/female)	3/18	4/18	0.120 (0.527)
Age (year)	72.4 ± 5.83 ^a^	71.0 ± 7.20	0.823 (0.466)
Height (cm)	154.0 ± 6.90	154.0 ± 8.30	0.728 (0.968)
Weight (kg)	60.4 ± 7.87	64.7 ± 12.50	1.790 (0.187)
BMI (kg/m^2^)	26.0 ± 3.24	26.3 ± 3.41	0.034 (0.752)

^a^ Mean ± standard deviation. CRYO = cryotherapy group, Non-CRYO = non-cryotherapy group.

**Table 2:** Changes in range of motion of the knee before and after the intervention (n = 43).

ROM		CRYO (n = 21)	Non-CRYO (n = 22)	t (*p*)
ROM-Flex (°)	Pre-	52.24 ± 9.19 ^a^	62.73 ± 12.20	-
	Post-	115.69 ± 9.58	100.75 ± 12.40	-
	Difference	−63.45 ± 15.99	−38.02 ± 16.29	5.167 (0.000) *
	t (*p*)	−18.185 (0.000)	−10.963 (0.000)	
ROM-Ext (°)	Pre-	11.76 ± 3.71	12.80 ± 4.14	-
	Post-	5.24 ± 1.09	6.23 ± 1.99	-
	Difference	6.25 ± 3.86	6.57 ± 4.40	0.035 (0.972)
	t (*p*)	7.755 (0.000) *	7.005 (0.000) *	

* *p* < 0.05, ^a^ Mean ± standard deviation. CRYO = cryotherapy group, Non-CRYO = non-cryotherapy group, ROM = range of motion, Flex = flexion, Ext = extension.

**Table 3:** Changes in pain before and after the intervention (n = 43).

PAIN		CRYO (n = 21)	Non-CRYO (n = 22)	t (*p*)
VAS	Pre-	5.05 ± 1.36 ^a^	4.90 ± 1.41	-
	Post-	2.00 ± 0.95	3.27 ± 1.67	-
	Difference	3.05 ± 1.50	1.64 ± 1.18	−3.442 (0.001) *
	t (*p*)	9.316 (0.000) *	6.521 (0.000) *	

* *p* < 0.05, ^a^ Mean ± standard deviation. CRYO = cryotherapy group, Non-CRYO = non-cryotherapy group, VAS = visual analog scale.

**Table 4:** Changes in swelling before and after the intervention (n = 43).

ROM		CRYO (n = 21)	Non-CRYO (n = 22)	t (*p*)
GIRTH-5 (cm)	Pre-	42.09 ± 4.04 ^a^	41.81 ± 4.40	
	Post-	38.40 ± 3.65	40.14 ± 5.08	
	Difference	3.68 ± 1.69	1.68 ± 1.64	−3.961 (0.000) *
	t (*p*)	10.007 (0.000)	4.796 (0.000)	
GIRTH-10 (cm)	Pre-	44.36 ± 4.62	43.54 ± 5.05	
	Post-	40.68 ± 3.96	41.87 ± 4.75	
	Difference	3.68 ± 1.95	1.66 ± 1.68	−3.618 (0.001) *
	t (*p*)	8.633 (0.000) *	4.406 (0.000) *	

* *p* < 0.05, ^a^ Mean ± standard deviation. CRYO = cryotherapy group, Non-CRYO = non-cryotherapy group.

**Table 5:** Changes in gait speed before and after the intervention (n = 43).

		CRYO (n = 21)	Non-CRYO (n = 22)	t (*p*)
10 MWT (m/s)	Pre-	20.85 ± 5.80 ^a^	19.52 ± 3.55	
	Post-	11.79 ± 2.53	14.81 ± 3.66	
	Difference	9.06 ± 5.25	4.70 ± 2.79	−3.422 (0.001) *
	t (*p*)	7.913 (0.000) *	7.922 (0.000) *	

* *p* < 0.05, ^a^ Mean ± standard deviation. CRYO = cryotherapy group, Non-CRYO = non-cryotherapy group, 10 MWT = 10 m walking test.

## Data Availability

The data presented in this study are available upon request from the corresponding author.

## References

[1] Farquhar S.J., Reisman D.S., Snyder-Mackler L. (2008). Persistence of altered movement patterns during a sit-to-stand task 1 year following unilateral total knee arthroplasty. Phys. Ther..

[2] Wang Y., Nguyen U.S.D., Lane N.E., Lu N., Wei J., Lei G., Zeng C., Zhang Y. (2021). Knee osteoarthritis, potential mediators, and risk of all-cause mortality: Data from the Osteoarthritis Initiative. Arthritis Care Res..

[3] Bijlsma J.W., Berenbaum F., Lafeber F.P. (2011). Osteoarthritis: An update with relevance for clinical practice. Lancet.

[4] Pang J., Cao Y.-L., Zheng Y.-X., Gao N.-Y., Wang X.-Z., Chen B., Gu X.-F., Yuan W., Zhang M., Liu T. (2015). Influence of pain severity on health-related quality of life in Chinese knee osteoarthritis patients. Int. J. Clin. Exp. Med..

[5] Strasser E.M., Draskovits T., Praschak M., Quittan M., Graf A. (2013). Association between ultrasound measurements of muscle thickness, pennation angle, echogenicity and skeletal muscle strength in the elderly. Age.

[6] Harato K., Otani T., Nakayama N., Watarai H., Wada M., Yoshimine F. (2009). When does postoperative standing function after total knee arthroplasty improve beyond preoperative level of function?. Knee.

[7] Sextro G.S., Berry D.J., Rand J.A. (2001). Total knee arthroplasty using cruciate-retaining kinematic condylar prosthesis. Clin. Orthop. Relat. Res..

[8] Gao J., Xing D., Dong S., Lin J. (2020). The primary total knee arthroplasty: A global analysis. J. Orthop. Surg. Res..

[9] Bade M.J., Kohrt W.M., Stevens-Lapsley J.E. (2010). Outcomes before and after total knee arthroplasty compared to healthy adults. J. Orthop. Sports Phys. Ther..

[10] Akeson W., Amiel D., Abel M., Garfin S., Woo S.L. (1987). Effects of immobilization on joints. Clin. Orthop. Relat. Res..

[11] Potter P.J., Kirby R.L., MacLeod D.A. (1989). The effects of simulated knee flexion contractures on standing balance. J. Biomech..

[12] Yun J.Y., Lee J.K. (2015). Effects of a thera-band exercise program on pain, knee flexion ROM, and psychological parameters following total knee arthroplasty. J. Korean Acad. Nurs..

[13] Piva S.R., Gil A.B., Almeida G.J., DiGioia A.M., Levison T.J., Fitzgerald G.K. (2010). A balance exercise program appears to improve function for patients with total knee arthroplasty: A randomized clinical trial. Phys. Ther..

[14] Kim I.-B., Kim Y.-S. (2009). The effect of PROM and AAROM exercise after TKA on increasing the knee range of motion. J. Korean Phys. Ther. Sci..

[15] Goh S.-L., Persson M.S., Stocks J., Hou Y., Welton N.J., Lin J., Hall M.C., Doherty M., Zhang W. (2019). Relative efficacy of different exercises for pain, function, performance and quality of life in knee and hip osteoarthritis: Systematic review and network meta-analysis. Sports Med..

[16] Chesterton L.S., Foster N.E., Ross L. (2002). Skin temperature response to cryotherapy. Arch. Phys. Med. Rehabil..

[17] Lee J.-H., Lee J.-H., Min D.-K., Lee J.-K., Kim J.-W. (2017). The Effects of Cryotherapy Treatment with Leg Elevation on Swelling of Patient Who had an TKA. J. Korean Acad. Orthop. Man. Phys. Ther..

[18] Lee J.G., Kim M.J., Park S.B., Kim Y.H. (1999). Changes in the Visual Analogue Scale Score Following Freezing Cold Air Application in Knee Joint Pain. J. Korean Acad. Rehabil. Med..

[19] Kim H.-B. (1988). Effect of Cryotherapy on Soft Tissue Injuries. J. Korean Phys. Ther. Assoc..

[20] Cameron M.H. (2012). Physical Agents in Rehabilitation: From Research to Practice.

[21] Choi Y., Jung B., Hwang B. (2013). Effects of skin temperature change, cold pain and muscle activity by Cold Air Application type on the induced delayed onset muscle soreness. J. Korean Soc. Radiol..

[22] Kim Y.-H., Yang G.-T., Jang Y.-H., Park S.-B., Ryu J.-S. (1998). The Development of a Cryotherapy System. J. Biomed. Eng. Res..

[23] Yoon A.J. (2014). Effect of Cryo Therapy after One-Side TKR on ESR, CRP, Pian and Swelling Over Time. Master’s Thesis.

[24] Hirvonen H., Mikkelsson M., Kautiainen H., Pohjolainen T., Leirisalo-Repo M. (2006). Effectiveness of different cryotherapies on pain and disease activity in active rheumatoid arthritis. A randomised single blinded controlled trial. Clin. Exp. Rheumatol..

[25] Bade M.J., Stevens-Lapsley J.E. (2011). Early high-intensity rehabilitation following total knee arthroplasty improves outcomes. J. Orthop. Sports Phys. Ther..

[26] Lee J., Min D., Lee S. (2020). The Effect of an Exercise Program with Patella Mobilization on Range of Motion, Muscle Strength and Gait in Patients with Total Knee Arthroplasty. J. Korean Soc. Integr. Med..

[27] Moon D.-H., Oh D.-H., Zhang S.-A., Lee J.-K. (2016). Effect of backward walking exercise on ROM, VAS score and proprioception in anterior cruciate ligament reconstruction patients. J. Korea Acad.-Ind. Coop. Soc..

[28] Huskisson E.C. (1974). Measurement of pain. Lancet.

[29] Shim E. (2014). A Study on the Effects of Structures Cryotherapy on Pain, Inflammation, Range of Motion and Edema in Patients with Total Knee Arthroplasty. Unpublished. Master’s Thesis.

[30] Choi Y., Seo K. (2014). Correlation among bioimpedance analysis, sonographic and circumferential measurement in assessment of breast cancer-related arm lymphedema. Lymphology.

[31] Wirz M., Van Hedel H.J., Rupp R., Curt A., Dietz V. (2006). Muscle force and gait performance: Relationships after spinal cord injury. Arch. Phys. Med. Rehabil..

[32] Kim Y.-K. (2002). The Effects of a 12-Week Exercise Training Program on Ligament Stability, Knee Function, and Lysholm Score after Anterior Cruciate Ligament Reconstruction.

[33] Lehmann J.F., Warren C.G., Scham S.M. (1974). Therapeutic heat and cold. Clin. Orthop. Relat. Res..

[34] Kullenberg B., Ylipää S., Söderlund K., Resch S. (2006). Postoperative cryotherapy after total knee arthroplasty: A prospective study of 86 patients. J. Arthroplast..

[35] Rowe P., Myles C., Walker C., Nutton R. (2000). Knee joint kinematics in gait and other functional activities measured using flexible electrogoniometry: How much knee motion is sufficient for normal daily life?. Gait Posture.

[36] Eller L.S. (1999). Guided imagery interventions for symptom management. Annu. Rev. Nurs. Res..

[37] Hwang H.S., Kim H.H., Shin J.W., Leem C.G., Lee C., Yang H.S., Lee D.M. (2004). Comparison of analgesic requirements for postoperative pain control in patients undergoing major orthopedic surgery. J. Korean Pain Soc..

[38] Yeo H.N., Kim Y.K., Kang M., Shin J.S. (2015). Effects of elastic band exercise on pain, range of motion, and fear of falling in patients with total knee replacement. J. Korean Clin. Nurs. Res..

[39] Chatap G., De Sousa A., Giraud K., Vincent J.-P., Acute Pain in the Elderly Study Group (2007). Pain in the elderly: Prospective study of hyperbaric CO_2_ cryotherapy (neurocryostimulation). Jt. Bone Spine.

[40] Karaduman Z.O., Turhal O., Turhan Y., Orhan Z., Arican M., Uslu M., Cangur S. (2019). Evaluation of the clinical efficacy of using thermal camera for cryotherapy in patients with total knee arthroplasty: A prospective study. Medicina.

[41] Hart J.M., Kuenze C.M., Diduch D.R., Ingersoll C.D. (2014). Quadriceps muscle function after rehabilitation with cryotherapy in patients with anterior cruciate ligament reconstruction. J. Athl. Train..

[42] Daniel D.M., Stone M.L., Arendt D.L. (1994). The effect of cold therapy on pain, swelling, and range of motion after anterior cruciate ligament reconstructive surgery. Arthrosc. J. Arthrosc. Relat. Surg..

[43] Carati C.J., Anderson S.N., Gannon B.J., Piller N.B. (2003). Treatment of postmastectomy lymphedema with low-level laser therapy: A double blind, placebo-controlled trial. Cancer Interdiscip. Int. J. Am. Cancer Soc..

[44] Mora S., Zalavras C.G., Wang L., Thordarson D.B. (2002). The role of pulsatile cold compression in edema resolution following ankle fractures: A randomized clinical trial. Foot Ankle Int..

[45] Webb J., Williams D., Ivory J., Day S., Williamson D. (1998). The use of cold compression dressings after total knee replacement: A randomized controlled trial. Orthopedics.

[46] Ohkoshi Y., Ohkoshi M., Nagasaki S., Ono A., Hashimoto T., Yamane S. (1999). The effect of cryotherapy on intraarticular temperature and postoperative care after anterior cruciate ligament reconstruction. Am. J. Sports Med..

[47] Saari T., Tranberg R., Zügner R., Uvehammer J., Kärrholm J. (2005). Changed gait pattern in patients with total knee arthroplasty but minimal influence of tibial insert design: Gait analysis during level walking in 39 TKR patients and 18 healthy controls. Acta Orthop..

[48] Okoro T., Ibrahim Y., Mansour N., Alderman P., Evans A. (2019). The use of cryotherapy in the early postoperative period after total hip arthroplasty. Ortop. Traumatol. Rehabil..

[49] Su E., Perna M., Boettner F., Mayman D., Gerlinger T., Barsoum W., Randolph J., Lee G. (2012). A prospective, multi-center, randomised trial to evaluate the efficacy of a cryopneumatic device on total knee arthroplasty recovery. J. Bone Jt. Surg. Br. Vol..

[50] Yoo M. (1995). New perspectives of treatment of osteoarthritis. J. Muscle Jt. Health.

